# Mechanisms of Action of Propofol in Modulating Microglial Activation in Ischemic Stroke

**DOI:** 10.3390/molecules30132795

**Published:** 2025-06-28

**Authors:** Pouria Abdolmohammadi, Bashir Bietar, Juan Zhou, Christian Lehmann

**Affiliations:** 1 Department of Microbiology and Immunology, Dalhousie University, Halifax, NS B2H 0A3, Canada; pouria.abdolmohammadi@dal.ca; 2 Department of Pharmacology, Dalhousie University, Halifax, NS B2H 0A3, Canada; bashir.bietar@dal.ca; 3 Department of Anesthesia, Pain Management and Perioperative Medicine, Dalhousie University, Halifax, NS B2H 0A3, Canada; juan.zhou@dal.ca

**Keywords:** stroke, inflammation, microglia, propofol

## Abstract

Ischemic stroke, responsible for the majority of stroke cases worldwide, triggers profound neuroinflammatory responses largely mediated by microglia. Excessive activation of pro-inflammatory microglia exacerbates neuronal injury, highlighting the need for therapeutic strategies targeting microglial modulation. Propofol (2,6-diisopropylphenol), a widely used intravenous anesthetic, has emerged as a promising neuroprotective agent due to its potent anti-inflammatory properties. This review comprehensively explores the diverse cellular mechanisms by which propofol attenuates microglial activation and inflammation in ischemic stroke. By elucidating these molecular pathways, it underscores the therapeutic potential of propofol in mitigating ischemic brain injury and guiding future clinical interventions.

## 1. Introduction

Ischemic stroke is the most common type of stroke, accounting for approximately 87% of all stroke cases [1,2]. In 2021, there were approximately 69.9 million cases of ischemic stroke globally, reflecting a 101.8% increase since 1990 [3]. The total number of deaths due to ischemic stroke was approximately 3.59 million in 2021. Multiple approaches have been explored to facilitate the repair of damaged neural networks and prevent disability due to ischemic stroke [4]. Ischemic stroke is caused by the blockage of a blood vessel supplying oxygen and nutrients to the brain, resulting in an immediate loss of function. The surrounding tissue forms a penumbra, an area deprived of oxygen and glucose. Although restoration of blood flow is the first line of treatment, it can cause secondary damage to the brain tissue, i.e., cerebral ischemia–reperfusion (I/R) injury [5].

The cells in the penumbra produce a variety of damage-associated molecular patterns (DAMPs), which are recognized by microglial pattern recognition receptors (PRRs), resulting in a robust release of pro-inflammatory cytokines and hence cytotoxicity [6]. Upon ischemic stroke, microglial polarization to a pro-inflammatory (M1) or anti-inflammatory (M2) state can determine the prognosis of stroke [7]. The excessive activation of M1 microglia in ischemic stroke is the main factor in terms of creating an hyper-inflammatory environment in the affected brain area, leading to neuronal injury and neurodegeneration [8,9,10,11]. Therefore, modulating microglial activation is important for alleviating inflammation during ischemic stroke [9,10].

**Figure 1:**
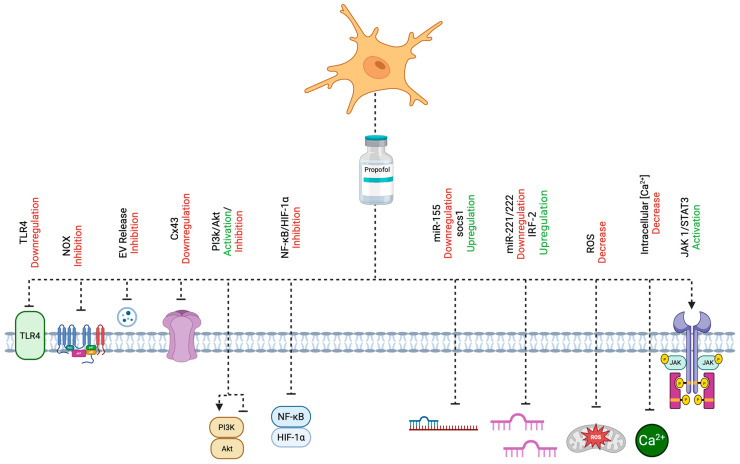
A comprehensive depiction of the cellular mechanisms through which propofol attenuates microglial activation and neuroinflammation in the context of ischemic stroke. Propofol exerts its neuroprotective effects via multiple molecular targets and signaling pathways, including the modulation of the PI3K/Akt cascade, inhibition of NOX, downregulation of TLR4 and Cx43, and activation of the JAK1/STAT3 pathway. It also regulates miRNA-mediated signaling, particularly the miR-155/SOCS1 and miR-221/222–IRF2 axes. Additional mechanisms include the suppression of the NF-κB/HIF-1α pathways, reduction in EV release, attenuation of oxidative stress via decreased ROS, and maintenance of intracellular Ca^2+^ homeostasis. Created with BioRender.com (URL accessed on 25 April 2025).

Propofol (2,6-diisopropylphenol) is an intravenous anesthetic widely used in clinical practice and is considered to have potentially anti-inflammatory properties in ischemic stroke via the modulation of microglial activation [11,12]. The main effect of propofol on the activation of microglia is supposed to be the inhibition of the release of proinflammatory cytokines throughout different mechanisms of action [13,14]. This review aims to describe the most important cellular mechanisms of action of propofol in ischemic stroke exerting neuroprotection. These molecular mechanisms are collectively illustrated in Figure 1, providing a visual summary of the pathways through which propofol modulates microglial activation and neuroinflammation in ischemic stroke.

## 2. Mechanisms of Action of Propofol

### 2.1. Target Receptors and Mechanisms Underlying Propofol’s Neuropharmacological Effects

The anesthetic effects of propofol are mainly mediated by the activation of GABA_A_ receptors—the major inhibitory receptors in mammalian brains—composed of five subunits that form a central chloride-ion-selective channel gated by the ligand, γ-aminobutyric acid (GABA) [15,16]. Propofol-induced GABA_A_ receptor activation results in an increase in the chloride influx current, contributing to the inhibition of glutamate release [17,18]. In addition, it has been found that propofol can inhibit glutamate release by blocking sodium currents through voltage-gated sodium channels [19].

The main anti-inflammatory propofol-related mechanisms of action are represented by the attenuation of the neurotoxic effects of excessive glutamate release through direct activation of the GABA_A_ receptors and inhibition of the N-methyl-d-aspartate (NMDA) receptors [20]. NMDA receptors play key roles in fast excitatory synaptic transmission and the production of a variety of proinflammatory cytokines [21,22]. It has been reported that propofol can inhibit NMDA receptors, leading to the attenuation of intracellular Ca^2+^ accumulation and, ultimately, a decline in the production of pro-inflammatory cytokines by microglia [23].

Propofol also initiates its anti-inflammatory effects via its lipophilicity [20]. Propofol as a lipid formulation integrates into cellular membranes, altering membrane structure and function, as well as ion-channel flow, second-messenger generation, and the production of cytokines and eicosanoids [20].

### 2.2. PI3K/Akt Pathway Activation and Inhibition

#### 2.2.1. PI3K/Akt Pathway Activation

Propofol has been shown to induce autophagy through PI3K/Akt pathway activation in a rat model of cerebral ischemia–reperfusion (I/R) injury and in oxygen–glucose deprivation (OGD)-activated primary microglia [24]. In general, the PI3K/Akt pathway has many vital biological functions for cells, including survival and growth under normal physiological conditions and in a variety of pathological disorders [24,25]. One of the essential outcomes by the PI3K/Akt pathway is the activation of autophagy in cells, which is crucial for recycling damaged organelles in injured cells and plays a key role in alleviating nerve injuries caused by stroke [24,26]. In a previous study, the effects of propofol on autophagy were investigated in rat models of cerebral ischemia–reperfusion (I/R) injury and in oxygen–glucose deprivation (OGD)-stimulated primary microglia derived from mouse brain cortex, with particular focus on the PI3K/Akt signaling pathway. To evaluate propofol-enhanced autophagy, the expression of LC3—a marker for autophagosome formation—was measured in rat brain tissue and primary microglial cells using immunofluorescence techniques. A dramatic increase in LC3 expression was observed upon propofol treatment [24]. To further confirm the enhancing effect of propofol on autophagy induction, they investigated the protein levels of the autophagy markers LC3II/I, Beclin-1, and Atg-7 using Western blot analysis; the results showed a significant increase in these protein levels in the I/R group treated with propofol compared to the untreated I/R group. They also measured the protein levels of OGD-activated primary microglia in vitro by Western blot and demonstrated that the levels of the LC3II/I, Beclin-1, and Atg-7 proteins were significantly increased in the propofol-treated primary microglia compared to the primary microglia that had not been treated with propofol [24]. This study also revealed a significant increase in the phosphorylation levels of PI3K, p-AKT, and p-mTOR proteins in both rat brain tissue with I/R injury and OGD-stimulated primary microglia, suggesting that propofol acts through the activation of the PI3K/Akt pathway [24]. Interestingly, in both rats with I/R injury and OGD-treated primary microglia, propofol caused the downregulation of a tumor suppressor gene PTEN, which inhibits the activation of the PI3K/Akt pathway [24]. Therefore, both the activation of the PI3K/Akt pathway and the downregulation of the PTEN gene are crucial for the protective role of propofol. Inhibition of the PI3K/Akt pathway with LY294002 prior to propofol treatment in I/R injury rats and OGD-stimulated primary microglia caused a significant reduction in autophagy-related markers, including LC3II/I ratio, Beclin-1, and Atg-7 [24]. This indicates that propofol upregulates autophagy through the PI3K/Akt pathway.

Another study showed the anti-inflammatory effects of delayed propofol delivery on the cytokine production of LPS-activated BV2 microglial cells through the PI3k/Akt pathway in different time intervals [27]. This study confirmed that activation of the PI3k/Akt pathway by propofol is key to reducing the production of inflammatory mediators, such as nitric oxide (NO), reactive oxygen species (ROS), and tumor necrosis factor (TNF) [27]. According to this study, BV2 cells treated with 50 μM propofol, showed a 2.1-fold increase in PKB phosphorylation at 30 min, rising to 2.3-fold after a 1 h incubation [27]. However, when the BV2 cell culture was treated with wortmannin—a PI3k/Akt pathway inhibitor—propofol’s effect on PKB phosphorylation was significantly reduced, simultaneously causing a significant elevation in the production of proinflammatory mediators NO, ROS, and TNF [27].

#### 2.2.2. PI3K/Akt Pathway Inhibition

Although activation of the PI3K/Akt pathway is involved in increasing autophagy in neurological disorders, other studies have posited that overactivation of the PI3K/Akt pathway and autophagy can also contribute to inflammation and cell death [28]. Thus, studying and understanding the downregulation of the PI3K/Akt pathway remains another important aspect in neurological diseases [29]. In microglia exposed to LPS and treated with propofol, it was shown that the miR-106b/Pi3k/Akt axis is an important pathway for inhibiting the activation of the microglia, thereby reducing the production of proinflammatory cytokines [29]. In this pathway, propofol upregulates the expression of miR-106, which promotes anti-inflammatory effects by downregulating the PI3k/Akt pathway [29]. In the same study, to further confirm the anti-inflammatory effects of propofol via miR-106, a loss-of-function (LOF) approach using miR-106 inhibitors was carried out. The results showed that inhibiting miR-106 expression completely abolished the anti-inflammatory effects of propofol, resulting in a marked increase in the expression levels of TIR domain-containing adaptor molecule 1 (Ticam1), myeloid differentiation primary response 88 (Myd88), interferon regulatory factor 3 (Irf3), and nuclear factor kappa B (NF-κB) transcripts [29]. These transcripts serve as key inflammatory indicators for assessing how propofol suppresses activation of the inflammatory signaling cascade—particularly the TLR-NF-κB axis—via miR-106 in the microglia [29]. This indicates that propofol can effectively inhibit the production of inflammatory mediators regulated via the NF-κB pathway [29]. To study the role of PI3k/Akt as the downstream pathway mediated by miR-106, an experiment using a PI3k/Akt pathway-specific agonist, 740Y-P, and antagonist, Wortmannin, was performed using Western blot. The levels of p-Akt were significantly upregulated when the cells were treated with 740Y-P and downregulated when the cells were treated with Wortmannin. After an upregulation in p-Akt by 740Y-P, the miR-106b-mediated inhibition of TNF and Nos2 was significantly reduced, indicating PI3k/Akt signaling as a key part of the miR-106b/PI3k/Akt axis [29].

### 2.3. Inhibition of Nicotinamide Adenine Dinucleotide Phosphate Oxidase

Inhibition of Nicotinamide Adenine Dinucleotide Phosphate (NADPH) oxidase (NOX) in overactivated microglia ameliorates inflammation [30]. NOX is a membrane-bound protein complex composed of multiple subunits, all of which play important functions in phagocytic cells [31]. In phagocytic cells such as the microglia, NOX takes part in the production of extracellular and intracellular reactive oxygen species (ROS) [31]. The complex of NOX is composed of cytoplasmic (p47^phox^, p67^phox^, p40^phox^, and Rac2) and membrane-bound (gp91^Phox^ and P22 ^Phox^) subunits [31]. When phosphorylated by particular kinases, the cytoplasmic subunits form a complex, translocating to the membrane to dock with the membrane subunits, contributing to the production of superoxide anions, which are precursors for ROS [31,32]. ROS produced by NOX regulate intracellular signaling and are also responsible for cell damage, such as neuronal damage in pathological conditions [33]. After a brain injury, such as a stroke, microglia start an uncontrolled massive production of ROS and proinflammatory cytokines, leading to neuroinflammation [34]. Upon ischemic stroke, NOX can be overactivated, leading to the excessive production of ROS, such as superoxide anion (O_2_^−^) and hydrogen peroxide (H_2_O_2_). This causes damage to the proteins, lipids, and nucleic acids of neural cells, and eventually contributes to their death [35,36]. In another study, in order to detect the anti-inflammatory effects of propofol by NOX inhibition, Luo et al. measured the enzymatic activity of NOX in LPS-stimulated BV2 microglia with or without propofol pretreatment [36]. The results showed that pretreatment with propofol significantly decreased NOX activity. The decrease in enzymatic NOX activity is due to the inhibition in the expression of the gp91^Phox^ and P22 ^Phox^ subunits, as shown by Western blot. Therefore, reduced assembly between the cytoplasmic and membrane subunits resulted in an inhibition of the production of proinflammatory factors in microglia [36]. In a parallel experiment, the authors found that the downregulation of gp91^Phox^ and P22 ^Phox^ expression by propofol is dose-dependent [36]. Furthermore, to assess the individual roles of the gp91^Phox^ and p22^Phox^ subunits, the authors used siRNA-mediated silencing of the corresponding messenger RNAs in BV2 microglia and found that silencing p22^phox^ reduced the anti-inflammatory effects of propofol, making it less effective at lowering nitric oxide and TNF production, whereas silencing gp91^phox^ did not significantly alter propofol’s anti-inflammatory effects [36]. All together, these results illustrate the anti-inflammatory effects of propofol by microglia inactivation via the inhibition of NOX [36].

### 2.4. Blocking and Downregulation of Toll-like Receptor 4 Expression

Toll-like receptor 4 (TLR4) is a pattern recognition receptor (PRR) that, upon binding to PAMPs or DAMPs, leads to immune responses by producing proinflammatory and anti-inflammatory cytokines following the activation of various types of signaling cascades [37,38,39]. After a stroke, the injured cells produce DAMPs that can be detected by TLR4s presented on microglia, leading to the production of proinflammatory cytokines. The uncontrolled release of inflammatory cytokines can worsen ischemic brain injury [40,41]. Following recognition of either PAMPs or DAMPs, TLR4 utilizes its Toll/interleukin-1 receptor (TIR) domain to recruit key adaptor proteins—including MyD88, MyD88-adapter-like protein (MAL, also known as Toll/Interleukin-1 receptor domain-containing adaptor protein, TIRAP), TIR-domain-containing adapter-inducing interferon-β (TRIF, also known as TIR domain-containing adaptor molecule-1, TICAM-1), and TRIF-related adaptor molecule (TRAM, also known as TIR domain-containing adaptor molecule-2, TICAM-2)—thereby inducing the downstream activation of transcription factors NF-κB, AP-1, and IRFs, contributing to the production of a variety of proinflammatory cytokines and finally inflammation [42,43,44,45]. According to some studies, propofol has been shown to play a key role in mediating inflammation by downregulating TLR4 [46,47]. In an MCAO model, propofol reduced TLR4 expression in microglia, resulting in a significant decrease in the mRNA expression of the proinflammatory cytokines IL-6, IL-β, and TNF [14]. Hence, downregulation of TLR4 in microglia, supressed proinflammatory cytokine production, and reduced infarct volume together contribute to a significant attenuation in brain injury in ischemic stroke [14]. This significant decrease in proinflammatory cytokine production was not observed in TLR4 knockout mice, demonstrating the anti-inflammatory effect of propofol in microglia through the downregulation of TLR4 [14].

In one study, the protective effect of propofol on OGD/OGD/R BV2 microglia via inhibiting TLR4/MyD88/NF-κB pathway was investigated [48]. Once microglial TLR4 binds to DAMPs, it can sequentially recruit MyD88, the interleukin-1 (IL-1) receptor-associated kinase, TNF receptor-associated factor 6 (TRAF6), and transforming growth factor-beta-activated kinase 1 (TAK1), resulting in the activation of the IkappaB kinase (IKK) complex [48]. When the IKK complex is activated, it phosphorylates IkappaB-α at serine residues 32 and 36, triggering its degradation. NF-κB is then released, translocated to the nucleus, and finally induces the transcription of kappaB-dependent genes, such as IL-1, IL-6, and TNF-α [48]. According to the Western blotting analysis obtained by Qin et al., an increase in the expression of TLR4 and MyD88 protein levels was observed when BV2 cells were exposed to OGD/R [48]. However, the TLR4 and MyD88 protein levels dramatically declined when the BV2 cells had been pretreated with propofol [48]. Furthermore, in order to further investigate the downstream pathway of TLR4-mediated signal transduction, the levels of IkappaB-α phosphorylation and IkappaB-α were measured with and without propofol treatment by Western blotting analysis [48]. The results showed that propofol significantly decreased the phosphorylation of IkappaB-α for BV2 cells under OGD/R conditions. Also, the NF-κB protein levels increased in the nucleus of OGD/R BV2 cells, whereas this was significantly reversed upon propofol pretreatment of OGD/R BV2 cells [48]. This corresponded with propofol markedly reducing TNF release from the OGD/R BV2 cells, as confirmed using ELISA.

In the same study, Qin et al. suggested that the inactivation of glycogen synthase kinase-3β (GSK-3β) can be another protective effect of propofol during stroke. Essentially, GSK-3β is a constitutively active serine/threonine kinase that becomes inactivated in TLR4-stimulated cells through the PI3K pathway by phosphorylation at the regulatory serine residue of position 9 (Ser9), resulting in a decrease in the proinflammatory cytokines and an increase in the anti-inflammatory cytokine IL-10 [49,50]. GSK-3β and p-GSK-3β protein expression in LPS-stimulated BV-2 microglia cells was detected by Western blotting, showing an increase of 3.2 fold in the ratio of p-GSK-3β to total GSK-3β. Pretreatment with propofol further increased the ratio by 1.4 fold. Thus, in TLR4-stimulated microglia, propofol can help enhance the phosphorylation of GSK-3β while also downregulating TLR4, resulting in a decrease in the proinflammatory cytokines and an increase in the anti-inflammatory cytokine IL-10 [50,51].

### 2.5. Downregulation of Connexin 43

Promoting a significant downregulation of Connexin 43 (Cx43) in microglia is another unique protective effect displayed by propofol during stroke [52,53]. Cx43 is a vertebrate protein, forming gap junction channels that conduct direct signaling between the cytoplasmic compartments of adjacent cells [54]. Moreover, Cx43 is also capable of forming hemichannels, which enable the release of factors and molecules such as ATP, glutamate, ions (like Ca^2+^), and other small proinflammatory or neurotoxic molecules into the extracellular medium [55,56,57]. Connexin 43 can be phosphorylated at various sites, which affects its assembly and function in different ways [58]. Upon ischemia, microglia is activated by proinflammatory cytokines, such as TNF and INF-gamma, increasing Cx43 expression [55,59]. Activated microglia migrate to compromised neurons, physically exchanging ions, second messengers, and small molecules throughout gap junction channels formed by Cx43, to promote proinflammatory and neurodegenerative cellular functions such as apoptosis in either microglia or neurons [52,59,60,61]. Furthermore, the debris and proinflammatory cytokines produced by the cells that underwent apoptosis can further promote an overexpression of Cx43 in microglia, contributing to abnormal and massive apoptosis signals that may even kill healthy cells [55,62]. In a study, the anti-inflammatory effects of propofol in stroke through the downregulation of microglial Cx43 was confirmed [52]. In the in vitro model (hypoxia/reoxygenation (H/R) injury), a significant 20% increase in the Iba1+ cells was observed using immunofluorescence staining after H/R injury, indicating an increase in microglial activation [52]. An increase in the level of proinflammatory cytokines—such as IL-1β, IL-6, and TNF—and a decrease in the level of anti-inflammatory cytokines—such as IL-10—was also observed [52]. Importantly, according to the Western blotting results, upon H/R exposure, an increase in the level of Cx43 and phosphorylated Cx43 (p-Cx43) in microglia was observed [52]. Moreover, using TUNEL assay, a dramatic increase in apoptosis among microglia cells was observed [52]. Furthermore, in an ex vivo model designed to study the role of H/R-injured microglia on neurons, rat primary neurons were cultured in the supernatant of H/R-injured microglia (microglia-conditioned supernatant, MCS) for 24 h. The results obtained by MTT assay showed that the viability of the neurons decreased slightly due to H/R injury, but a significant decrease was observed after culture with MCS [52]. Using 40 μM propofol treatment, microglial viability improved, and Iba1, Cx43, Cx43, and p-Cx43 expression decreased, illustrating that propofol feasibly attenuates the expression and phosphorylation of Cx43 and decreases microglia activation [52]. Moreover, a noticeable decrease in the proinflammatory cytokines such as IL-1β, IL-6, and TNF, along with an increase in the anti-inflammatory cytokines such as IL-10 was observed, meaning propofol treatment could significantly protect the microglial cells from the effects of H/R injury [52]. Thus, the results confirmed that propofol has anti-inflammatory effects against I/R injury via the downregulation of Cx43 in microglial cells, thereby decreasing neuronal apoptosis [52]. In order to investigate the role of Cx43 in propofol rescue on H/R-induced neuronal impairment, they knocked down microglial Cx43. The silencing of Cx43 combined with propofol treatment resulted in a significant reduction in microtubule-associated protein 2 (Map2) expression and a higher morphological recovery among neurons treated with H/R-exposed MCS, compared with propofol treatment without Cx43 knock down [52]. Also, upon Cx43 knock down and propofol treatment, a more dramatic increase in the viability of microglial cells was also observed, along with a decrease and an increase in proinflammatory and anti-inflammatory cytokines, respectively, when compared to the H/R-injured cells. Finally, they studied the protective effect of propofol in I/R injury in an in vivo middle cerebral artery occlusion (MCAO) rat model of stroke [52]. The results were consistent with the data obtained from in vitro results, as MCAO animals with propofol treatment and the downregulation of Cx43 showed a significant decrease in cerebral infarct volume and neuronal apoptosis [52]. Through an overexpression of Cx43, the I/R injury conditions deteriorated, although propofol could still slightly attenuate cell death and microglial activation via downregulation of Cx43 expression [52].

### 2.6. JAK1/STAT3 Pathway Activation

The activation of specific Janus kinase-signal transducers and activators of transcription (JAK/STAT) signaling pathway such as JAK1/STAT3, by propofol has been reported to be neuroprotective in stroke [63,64]. The JAK/STAT pathway is engaged in both the immune response of a variety of cytokines and the actions of non-immune mediators such as growth factors and hormones [65]. In fact, the JAK family consists of four members (JAK1, JAK2, JAK3, and tyrosine kinase 2 (TYK2)) and the STAT family consist of seven members (STAT1, STAT2, STAT3, STAT4, STAT5a, STAT5b, and STAT6) [66]. JAK members all have different receptor affinities that are constitutively associated with their cytoplasmic portions [66,67]. When a specific ligand binds to its receptor, an active receptor complex is assembled, in which its cytoplasmic portion phosphorylates the receptor-associated JAKs. In turn, the phosphorylation of JAKs provides docking sites for STATs that as a result become phosphorylated on their tyrosine and serine residues [65,68]. Then, phosphorylated STATs are released from the receptor complex, form dimers, translocate to the nucleus to bind to the promoter regions of specific target genes, leading to the regulation of the transcription of these genes [65,67,68]. Among the JAK/STAT family members, activation of the JAK1/STAT3 pathway has shown anti-inflammatory effects in stroke [63,69]. Basically, the phosphorylation of JAK1 can regulate STAT3 activity via phosphorylating STAT3 on the tyrosine705 and serine727 residues, in which reduced phosphorylation at Ser727 residue is usually correlated with an increase in the phosphorylation of Tyr705 residue [63,70,71]. In a previous study, CoCl_2_-induced, hypoxia-injured BV2 cells were used to establish an in vitro hypoxia model to evaluate the protective effects of propofol in ischemic/hypoxic stroke [63]. According to the results obtained by Western blot, CoCl_2_ treatment decreased the expression and phosphorylation of JAK1 [63]. Moreover, CoCl_2_ treatment reduced the phosphorylation of STAT3 at Tyr-705, with no effect on the phosphorylation of STAT3 at Ser-727 [63]. However, propofol pretreatment diminished these CoCl_2_-modulated effects in CoCl_2_-induced hypoxic injured BV2 cells [63]. Additionally, in order to further investigate the anti-inflammatory effects of propofol via the activation of the JAK1/STAT3 pathway, Lu et al. pretreated BV2 cells with propofol and selective JAK1 inhibitor INCB039110, followed by CoCl_2_ treatment [63]. According to the results, INCB039110 abolished the effect of propofol, leading to an increase in the production of TNF [63]. Although it is evident that propofol’s anti-inflammatory effect in stroke involves activation of the JAK1/STAT3 pathway through increased JAK1 phosphorylation and the subsequent phosphorylation of STAT3 at Tyr-705, the exact molecular mechanisms by which propofol initiates or regulates this pathway remain unclear [63].

Furthermore, some studies suggest that STAT3 can function as both a proinflammatory and an anti-inflammatory factor, depending on its activation context and phosphorylation status. In the anti-inflammatory context, STAT3 promotes M2 microglial polarization, which is associated with tissue repair and anti-inflammatory responses. However, under certain conditions, STAT3 activation can also support proinflammatory processes, especially if the degree or pattern of phosphorylation is altered, for example, aberrant or excessive activation may promote inflammatory gene expression [72,73]. So, while the JAK/STAT3 pathway is involved in promoting M2 polarization and anti-inflammatory effects, the pathway itself is versatile because STAT3’s effects are context-dependent and can shift toward either anti-inflammatory or proinflammatory outcomes, depending on how precisely it is regulated [72,73].

### 2.7. miR-155/SOCS1 Pathway

Some anti-inflammatory effects of propofol have been associated with the downregulation of specific miRNAs such as miR-155 [74]—which is expressed upon the activation of TLR4 and represents one of the most important miRNAs regulating central nervous system (CNS) inflammatory responses—and is crucial for the robust induction of some proinflammatory cytokine genes such as IL-6 and TNF in microglia [75,76,77]. At the same time, upon microglia activation by LPS through TLR4, some negative feedback loops—such as the suppression of cytokine signaling-1 (SOCS1) expression—are initiated in order to prevent hyperresponsiveness and develop endotoxin tolerance [78]. It has been reported that miR-155 proinflammatory function comes by downregulating SOCS1 in microglia [79,80,81]. SOCS1 is a protein that inhibits cytokine signal translation by the direct inhibition of JAK/STAT activation, leading to an inhibition in the production of cytokines [79,82,83]. Thus, miR-155 post-transcriptionally regulates SOCS1 by targeting and degrading SOCS1 mRNA [79]. In another study, LPS-activated BV2 microglia were used in order to investigate the anti-inflammatory effects of propofol via the inhibition of miR-155/SOCS1 [74]. According to the results obtained by PCR assay, treating the BV2 cells with an increasing concentration of LPS contributed to a significant dose-dependant expression of miR-155 [74]. However, when the LPS-activated BV2 microglial were treated with propofol, a significant decrease in miR-155 expression was observed. To further evaluate the role of miR-155 in the anti-inflammatory effect of propofol, an miR-155 inhibitor was used to knock down miR-155. According to the results, in un-transfected BV2 cells, the production of proinflammatory mediators and cytokines such as NO, TNF, and IL-6 significantly decreased by propofol [74]. However, in the miR-155 knockdown BV2 cells, LPS induction of the proinflammatory cytokines were less robust, and propofol had very little effect on the production of these cytokines, indicating the critical role of miR-155 in the anti-inflammatory effect of propofol [74]. Moreover, the protein expression of SOCS1 in different treatments was measured [74]. In the cells transfected with a negative control inhibitor—a non-targeting inhibitor used as a baseline reference to compare against the specific inhibition of miR-155—the protein expression of SOCS1 was slightly increased upon LPS treatment [74]. However, the SOCS1 protein expression was significantly higher in the presence of propofol and LPS than LPS alone, confirming that propofol can upregulate SOCS1 [74]. Although propofol dramatically increased SOCS1 in negative control inhibitor-transfected cells upon LPS stimulation, it failed to induce SOCS1 in miR-155 knockdown cells [74]. Furthermore, in order to confirm the SOCS1 role in anti-inflammatory effects of propofol, the nitrite and cytokine levels in SOCS1downregulated microglial cells were measured [74]. BV2 microglial cells were transfected with either SOCS1 or control siRNA [74]. According to the results, in SOCS1 knockdown cells, the production levels of NO, TNF, and IL-6 were significantly increased in response to LPS compared to the control siRNA [74]. However, propofol markedly reduced the LPS-induced production of NO, TNF, and IL-6 in the control siRNA-treated cells—but not as significantly in SOCS1 knockdown cells—indicating that SOCS1 plays an essential role in the anti-inflammatory effects of propofol [74]. Overall, it can be concluded that the anti-inflammatory effects of propofol through the miR-155/SOCS1 pathway result from downregulating miR-155 and, consequently, upregulating SOCS1 expression—altogether contributing to a significant decrease in the production of proinflammatory mediators [74].

### 2.8. miR-221/222-IRF2 Pathway

The miR-221/222-IRF2 axis is considered as an anti-inflammatory pathway activated by propofol [84]. The miR-221/222 gene cluster is located on chromosome Xp11.3 [85,86]. The promoter region of miR-221/222 contains two canonical TATA boxes located 550 and 190 base pairs upstream of pre-miR-222, and three poly-A sequences downstream of pre-miR-221 [86,87]. Angiotensin II regulates the expression of this gene cluster along with a repressive complex, including estrogen receptor α and the nuclear receptors NCOR1 and NCOR2 [88,89]. miR-221 and miR-222 are encoded and transcribed together as pri-miR, with the two paralogous miRs separated by 726 bp and sharing the same seed nucleotide sequence [86,90]. Throughout nuclear processing mediated by Drosha and RNA-binding protein DiGeorge syndrome critical region gene 8 (DGCR8), the pri-miR transcription generate 110-nucleotide pre-miR-221 and pre-miR-222 [86]. The other component of the miR-221/222-IRF2 pathway, interferon regulatory factor 2 (IRF2), acts as a transcriptional repressor by binding to the same DNA sequence as IRF1—a pro-inflammatory transcription factor—does [91,92]. IRF2 exerts its transcriptional suppression effect by interacting with co-repressors, including IRF2 binding protein 2 (IRF2BP2), resulting in the activation of anti-inflammatory (M2) marker genes and the suppression of pro-inflammatory (M1) marker genes [91,93]. Some studies have shown that miR-221/222 reduces IRF2 protein levels by inhibiting IRF2 translation, thereby contributing to inflammation [91,93]. However, other studies have shown that propofol counteracts miR-221/222 function, which in turn increases IRF2 protein levels, leading to a decrease in the level of proinflammatory cytokines [84]. This study reported that the LPS-induced upregulation of miR-221/222 was markedly abolished by propofol treatment [84]. In order to investigate the role of miR-221/222 in microglia activation, they overexpressed miR-221/222 in BV2 cells by transfecting them with miR-221 and miR-222 mimics [84]. The results revealed a significant increase in the production of IL-1β, IL-6, and TNF-α in the cell culture supernatants [84]. However, when they silenced miR-221/222 expression in the LPS-primed BV2 cells via miR-221 and miR-222 inhibitors, they observed a dramatic decrease in the inflammatory cytokine levels, indicating the regulatory role of miR-221/222 in microglia activation [84]. In addition, using three publicly available algorithms (TargetScan, miRDB, and miRBase) it was found that IRF gene was the target of miR-221/222. In fact, ectopic expression in miR-221 or miR-222 caused a reduction in IRF2 protein expression in BV2 cells [84]. Finally, it was examined whether the miR-221/222-IRF2 axis mediates the anti-inflammatory role of propofol in microglia activation. Xiao et al. upregulated miR-221/222 in LPS-stimulated BV2 cells using propofol treatment [84]. The results showed a significant decrease in the expression of inflammatory genes (Il1b, IL6, Tnf, Ptgs2, and Nos2) and hence the level of cytokines due to propofol treatment, which could be restored by either miR-221 or miR-222 mimics [84]. Moreover, the genetically silenced IRF2 in LPS-primed BV2 cells with propofol treatment confirmed propofol’s failure to induce an inhibitory effect [84]. All these findings indicate that the miR-221/222–IRF2 axis is an essential functional mediator of propofol in suppressing microglia activation [84].

### 2.9. NF-κB/Hif-1α Signaling Pathway

Inhibition of the NF-κB/Hif-1α signaling pathway represents another mechanism by which propofol exerts its anti-inflammatory effects in neuroinflammation [94]. The NF-κB family of transcription factors include five members—p50, p52, p65 (also known as RelA), c-Rel, and RelB—that exist as either hetero- or homo-dimeric complexes [95,96]. These subunits are inactivated in the cytoplasm by the members of the IkB family [95]. Upon activation by certain compounds such as TNF, a kinase signaling cascade is induced, resulting in the IKK-mediated phosphorylation of IkB and its subsequent poly-ubiquitin-mediated proteasomal degradation. This contributes to the release of NF-kB, which translocates into the nucleus and binds to target gene promoters and enhancers, contributing to the production of a variety of proinflammatory cytokines [95,97,98,99]. Importantly, it has been reported that NF-κB subunit p65 plays a key role in the production of Hif-1α mRNA and protein [100,101]. Hypoxia inducible factor-1α (Hif-1α) is a subunit of HIF-1, a transcription factor that is initiated under low-oxygen conditions [95,102]. In fact, the stability of the Hif-1α subunit is improved during hypoxia, which causes Hif-1α to upregulate the transcription of proinflammatory genes, such as cytokines [103,104]. According to many studies, NF-kB plays a key role as the transcriptional activator of Hif-1α; in the absence of NF-kB, the Hif-1α gene is not transcribed, even during a lengthened hypoxia [101,105,106]. Propofol can supress the expression of Hif-1α by downregulating NF-κB p65 in CoCl_2_ hypoxic-induced BV2 cells, thereby inhibiting the production of proinflammatory cytokines TNF, IL-1β, and IL-6 [94]. In this study, in order to examine the anti-inflammatory effects of propofol via inhibiting the NF-κB/Hif-1α pathway, the authors measured the levels of the two proteins—NF-κB p65 and Hif-1α—that propofol potentially affects [94]. The cells were pretreated with propofol about 3 h prior to CoCl_2_ stimulation for 24 h, and then the levels of NF-κB p65 and Hif-1α production were measured by Western blotting [94]. The results revealed that propofol dramatically reduced the production of NF-κB p65 and Hif-1α compared to the CoCl_2_-treated group [94]. In order to further investigate the role of NF-κB in propofol-related anti-inflammatory mechanisms, the authors treated BV2 cells with siRNA against NF-κB p65, followed by exposure to hypoxia and incubation for 24 h [94]. According to the results, in NF-κB p65-silenced and CoCl_2_-treated cells, the levels of Hif-1α and IL-1β were downregulated compared to those in only CoCl_2_-treated cells [94]. Thus, propofol inhibits the upregulation of NF-κB p65, which in turn suppresses Hif-1α production, resulting in a decrease in the hypoxia-induced inflammation in BV2 cells [94].

### 2.10. Extracellular Vesicle Release

More recently, another anti-inflammatory mechanism of propofol has been reported, i.e., the inhibition of extracellular vesicle release [107]. Extracellular vesicles (EVs) are membrane-enclosed cargos—including exosomes and microvesicles—which are key players in intercellular signaling [108,109]. Upon brain damage, microglial cells release these vesicles to secrete proinflammatory cytokines, leading to inflammation [108,110,111]. EV release by microglia is generally stimulated by exposure to immune activators such as ROS, ATP, LPS, and TNF [107,111,112]. In a study performed using LPS-stimulated BV2 cells, the anti-inflammatory effects of propofol through the downregulation of immune-activated EV release was confirmed [107]. In this study, using Western blotting, the authors first measured EV release from LPS-stimulated microglia in the presence and absence of propofol by determining the levels of EV markers flotillin-2 and tissue transglutaminase (tTG) in protein lysates from the EV pellets [107]. According to the results, LPS alone significantly increased the protein levels of flotillin-2 and tTG; however, propofol treatment dramatically decreased flotillin-2 and tTG levels, indicating that propofol could decrease EV release from microglia [107]. Furthermore, in order to investigate the role of EVs on propofol-mediated anti-inflammatory response in microglia, the authors examined whether the anti-inflammatory effects of propofol could be reversed by the addition of EVs isolated from immune-activated microglia to the treatment groups, along with LPS and propofol [107]. The results revealed that the downregulation of the M1 marker genes and the upregulation of the M2 marker gene mediated by propofol was completely reversed upon EV treatment, confirming that propofol exerts its anti-inflammatory effects in activated microglia through a reduction in EV release [107]. In addition, to measure the reduction in microglia-mediated neurotoxicity through EV release inhibition by propofol, they collected EVs from LPS-stimulated BV2 cells and added them to the experimental groups [107]. According to the results, EV treatment did not markedly influence microglia-mediated neurotoxicity toward N2A cells in the absence of LPS [107]. However, EV treatment dramatically reversed propofol-mediated neuroprotection by LPS-stimulated microglia [107]. Finally, MAP2 ELISA was used in order to more specifically target MAP2-positive neurons in N2A cultures and quantitatively determine neuronal survival upon LPS and propofol treatment [107]. Propofol reversed the neurotoxicity caused by LPS-stimulated microglia in conditioned medium, although such protection was abolished by the addition of EVs to microglia cultures before collection of the conditioned medium [107]. LPS-activated microglia also induced TUNEL-positive cells in the cultures, which is a sign of apoptosis, and this effect was further exacerbated by the addition of EVs to microglia cultures before the collection of the conditioned medium. In summary, the results confirmed that propofol pretreatment decreased activated microglia-mediated neurotoxicity by inhibiting EV release [107].

### 2.11. Oxidative Stress and Increasing Antioxidant Activity

Mitochondria, as double-membrane subcompartments of cells, are a major source of ROS production, alongside their critical role in ATP production [113,114]. After stroke and IR injury, elevated levels of ROS produced by mitochondrial dysfunction contribute to oxidative stress, resulting in neurodegeneration [113,115,116,117]. Although the causes of mitochondrial ROS production during reperfusion remain unclear, it has been reported that elevated levels of ROS production by mitochondria during hypoxia-induced stroke and IR injury can be due to abnormally high levels of succinate, which is a citric acid cycle intermediate molecule in mitochondria [115,118]. Moreover, the overproduction of ROS during IR injury can also cause a decrease in mitochondrial membrane potential, thereby aggravating mitochondrial dysfunction [94]. In a study performed using CoCl_2_ hypoxic-induced BV2 cells, the protective effects of propofol through ameliorating oxidative stress and increasing antioxidant activity were confirmed [94]. According to the results, CoCl_2_ induced higher ROS production in BV2 cells compared to control cells, while propofol decreased ROS levels compared to CoCl_2_-treated cells [94]. At the same time, CoCl_2_ decreased superoxide dismutase (SOD) activity, and total antioxidant capacity (T-AOC), whereas propofol ameliorated this reduction [94]. The restoration of antioxidant activity SOD and T-AOC by propofol inhibits ROS overproduction and inflammatory responses [94]. SOD restoration by propofol can inhibit the production of inflammatory responses via the inhibition of NF-κB activation [94,119]. Moreover, ROS reduction by propofol can inhibit Fe^2+^ to Fe^3+^ oxidation, which in turn prevents Hif-1α protein stabilization, leading to a downregulation of inflammatory mediators [94]. Propofol also ameliorated the decrease in mitochondrial membrane potential in CoCl_2_-treated microglia [94].

### 2.12. Intracellular Ca^2+^ Homeostasis

An important anti-inflammatory mechanism of propofol in microglia has been reported to be the maintenance of intracellular Ca^2+^ homeostasis [63]. Many of the microglia’s physiological functions—such as cell proliferation, differentiation, migration, and the induction of intracellular enzymatic pathways involved in the transcriptional regulation of many genes—have been known to be linked to intracellular Ca^2+^ signaling [120,121]. According to some studies, an increase in the microglial intracellular Ca^2+^ concentration is related to the production of proinflammatory cytokines and ROS [122,123]. According to these studies, although an increase in the microglial intracellular Ca^2+^ concentration is required for the production of inflammatory cytokines, it is not sufficient on its own; it is also necessary to treat the microglia cells with LPS [122,123]. In nervous tissue, CaMKIIα is the major isoform of Ca^2+^/calmodulin-dependent protein kinase (CaMK), which is highly sensitive to intracellular Ca^2+^ levels, and its activation is associated with the production of proinflammatory cytokines, such as TNF and IL-1β [63,124,125]. Moreover, ERK 1/2 and NF-κB are also involved in Ca^2+^-mediated TNF-α release [63]. In a study performed using CoCl_2_ hypoxic-induced BV2 cells, the anti-inflammatory effects of propofol through maintaining intracellular Ca^2+^ homeostasis was studied [63]. According to the results, compared to the control, CoCl_2_ treatment increased the cytoplasmic Ca^2+^ concentration, along with the phosphorylation of the CAMKIIα, ERK, NF-κB proteins [63]. However, propofol pretreatment attenuated the increase in CoCl_2_-induced intracellular Ca^2+^ levels and downregulated the phosphorylation of CAM-KIIα, ERK, and NF-κB [63]. In order to investigate the role of Ca^2+^ homeostasis and the phosphorylation of CAMKIIα, ERK, and NF-κB in TNF production, BV2 cells were pretreated with calcium chelator BAPTA-AM, CAMKIIα inhibitor KN93, or ERK inhibitor U0126, followed by CoCl_2_ treatment. The results revealed that BAPTA-AM, KN93, and U0126 dramatically decreased TNF production, similar to the effect of propofol [63]. To confirm the role of the phosphorylation of CAMKIIα, ERK, and NF-κB in the protective effects of propofol against CoCl_2_ treatment, BV2 cells were pretreated with calcium chelator BAPTA-AM, CAMKIIα inhibitor KN93, or ERK inhibitor U0126, followed by CoCl_2_ treatment [63]. According to the results, similar to propofol treatment, BAPTA-AM and KN93 significantly decreased the phosphorylation of CAMKIIα [63]. Furthermore, BAPTA-AM, KN93, and U0126 markedly decreased the phosphorylation of ERK and NF-κB, also similar to propofol treatment [63]. In addition, they found that CoCl_2_ treatment increased apoptosis and the expression of cleaved caspase 3 in the cells; however, these effects were inhibited by propofol treatment, suggesting that apoptosis induced by CoCl_2_ could be inhibited by propofol [63]. Finally, BV2 cells were pretreated with calcium chelator BAPTA-AM, CAMKIIα inhibitor KN93, or ERK inhibitor U0126, followed by CoCl_2_ treatment, in order to investigate the role of Ca^2+^ homeostasis and the phosphorylation of the CAMKIIα, ERK, and NF-κB pathways in cell apoptosis [63]. According to the results, BAPTA, KN93, and U0126 could each reduce the percentage of apoptotic cells [63]. In addition, it was found that BAPTA, KN93, and U0126 could each inhibit the expression of cleaved caspase 3. However, propofol, BAPTA-AM, KN93, and U0126 pretreatment showed no effect on the expression of pro-caspase 3 [63]. Overall, the results revealed that one of the anti-inflammatory mechanisms of propofol is via limiting intracellular cellular Ca^2+^ overload, modulating the phosphorylation of CaMKIIα, ERK, and NF-κB, resulting in a decrease in the proinflammatory cytokine production and cell apoptosis.

All in all, a comprehensive summary of the experimental models, methodologies, and key findings supporting these mechanistic pathways is presented in Table 1.

## 3. Materials and Methods

A literature search was conducted to identify studies investigating the immunomodulatory and neuroprotective effects of propofol, with a focus on microglial activation and ischemic stroke. The search was performed using PubMed, employing the following keywords: “propofol,” “microglia,” “neuroinflammation,” “ischemic stroke”. Studies were included if they were original research articles published in English, investigated propofol’s effects on microglia or inflammation-related mechanisms, and used in vitro, in vivo, or ex vivo experimental models relevant to central nervous system inflammation. Exclusion criteria included studies not involving microglia or not focused on inflammation-related pathways, and articles without full-text access.

## 4. Conclusions

Ischemic stroke remains a major global health challenge, with neuroinflammation playing a critical role in secondary brain injury. Microglia, as central mediators of the inflammatory response, represent a crucial therapeutic target for limiting neuronal damage and promoting recovery. Propofol, beyond its well-established anesthetic properties, has demonstrated significant anti-inflammatory and neuroprotective effects in experimental models of stroke, primarily through its ability to modulate microglial activation. The evidence summarized in this review highlights the multifaceted ways in which propofol can attenuate inflammation and mitigate ischemic injury.

Numerous studies support the anti-inflammatory and neuroprotective properties of propofol in stroke models; however, a critical comparison reveals several gaps and inconsistencies. For instance, although PI3K/Akt activation is associated with autophagy-mediated neuroprotection, some reports indicate that excessive activation of this pathway may contribute to inflammation, suggesting that the context and degree of activation are crucial in determining its effects. Additionally, many in vitro studies rely on BV2 microglial cell lines or hypoxia mimetics, such as CoCl_2_, which may not accurately replicate the complexity of in vivo ischemic stroke environments. Few investigations employ standardized dosing protocols or the pharmacokinetic modeling of propofol, and many omit assessments of long-term behavioral or functional outcomes. Moreover, critical biological variables such as sex, age, and stroke severity are often not considered, despite their known influence on inflammatory responses and recovery trajectories. These limitations underscore the need for more rigorous, standardized, and translationally relevant experimental models to better evaluate the therapeutic potential of propofol and guide its clinical application in ischemic stroke.

Although the preclinical findings are promising, clinical studies are essential to validate the translational potential of propofol as an adjunct therapy in stroke management. This includes exploring optimized dosing, timing, and safety in human subjects. Bridging this gap will require rigorously designed clinical trials that incorporate stratification by patient age, sex, and stroke characteristics to determine whether the anti-inflammatory benefits observed in experimental models can be effectively and safely translated into human stroke therapy. Understanding and optimizing the therapeutic application of propofol could offer new avenues for improving outcomes in patients suffering from ischemic stroke.

## Figures and Tables

**Table 1:** Summary of experimental models and outcomes for the anti-inflammatory effects of propofol in microglia via different mechanisms of action.

Mechanism	Type of Experiment	Model	Outcome	Reference
PI3K/Akt/Pathway Activation	in-vivo	Rat I/R Injury	Autophagy Activation (Anti-inflammatory Effects)	[24]
PI3K/Akt/Pathway Activation	in-vitro	(OGD)-Stimulated Primary Microglia	Autophagy Activation (Anti-inflammatory Effects)	[24]
PI3K/Akt/Pathway Activation	in-vitro	LPS-induced BV-2 microglia	Anti-inflammatory Effects	[27]
PI3K/Akt/Pathway Inhibition	in-vitro	LPS-induced Primary Mouse Microglia	Anti-inflammatory Effects	[29]
NADPH oxidase Inhibition	in-vitro	LPS-induced BV2 Cells	Anti-inflammatory Effects	[36]
Downregulation of TLR4 Expression	in-vivo	(MCA) coagulation	Anti-inflammatory Effects	[14]
Downregulation of TLR4 Expression	in-vitro	(OGD/R) BV2 microglia	Anti-inflammatory Effects	[48]
Downregulation of TLR4 Expression, but maintaining GSK-3β	in-vitro	LPS-induced BV-2 microglia	Anti-inflammatory Effects	[51]
Downregulation of Connexin 43	in-vitro	hypoxia/reoxygenation-H/R injury	Anti-inflammatory Effects	[52]
Downregulation of Connexin 43	ex-vivo	MCS was collected from H/R-injured microglia.	Anti-inflammatory Effects	[52]
Downregulation of Connexin 43	in-vivo	Middle cerebral artery occlusion (MCAO) in SD rats	Anti-inflammatory Effects	[52]
Activation of JAK1/STAT3 pathway	in-vitro	CoCl_2_-induced hypoxic injured BV2 cells	Anti-inflammatory Effects	[63]
Regulating the miR-155/SOCS1 Pathway	in-vitro	LPS-induced BV-2 microglia	Anti-inflammatory Effects	[74]
Regulating MicroRNA-221/222-IRF2 Pathway	in-vitro	LPS-induced BV-2 microglia	Anti-inflammatory Effects	[84]
Inhibiting NF-κB/Hif-1α Pathway	in-vitro	CoCl_2_ hypoxic-induced BV2 cells	Anti-inflammatory Effects	[94]
Inhibiting Extracellular Vesicle Release	in-vitro	LPS-induced BV-2 microglia	Anti-inflammatory Effects	[107]
Inhibiting ROS and Increasing Antioxidant Activity	in-vitro	CoCl_2_ hypoxic-induced BV2 cells	Anti-inflammatory Effects	[94]
Maintaining Intracellular Ca^2+^ Homeostasis	in-vitro	CoCl_2_ hypoxic-induced BV2 cells	Anti-inflammatory Effects	[63]
